# Health-related quality of life and its associated factors among hemophilia patients: experience from Ethiopian Hemophilia Treatment Centre

**DOI:** 10.1186/s40780-023-00326-6

**Published:** 2024-01-02

**Authors:** Sitina Iyar, Girma Tekle Gebremariam, Dessale Abate Beyene, Amha Gebremedhin, Tamrat Assefa Tadesse

**Affiliations:** 1 Department of Pharmacology and Clinical Pharmacy, School of Pharmacy, College of Health Sciences, Addis Ababa University, Addis Ababa, Ethiopia; 2 Department of Pharmacy, Asrat Woldeyes Health Science Campus, Debre Berhan University, Debre Berhan, Ethiopia; 3 Department of Internal Medicine, School of Medicine, College of Health Sciences, Addis Ababa, University, Addis Ababa, Ethiopia

**Keywords:** Hemophilia, HRQoL, EQ 5D 5L, EQ-VAS, Ethiopia

## Abstract

**Background:**

Hemophilia is a rare genetic condition that is often overlooked and underdiagnosed, particularly in low-income countries. Long-term spontaneous joint bleeding and soft tissues can have a significant negative impact on a patient’s health-related quality of life (HRQoL). The objective of this study was to assess HRQoL and its associated factors in Ethiopian patients with hemophilia.

**Methods:**

A cross-sectional survey was conducted among patients with hemophilia at Tikur Anbessa Specialized Hospital (TASH) in Addis Ababa, Ethiopia. Patients were recruited consecutively during follow-up visits. The European Quality of Life Group’s 5-Domain Questionnaires at five levels (EQ-5D-5L) and Euro Quality of Life Group’s Visual Analog Scale (EQ-VAS) instruments were used to assess HRQoL. The EQ-5D-5L utility score was computed using the disutility coefficients. We applied the Krukal-Wallis and Mann–Whitney U tests to determine the differences in EQ-5D-5L and EQ-VAS utility scores between patient groups. A multivariate Tobit regression model was used to identify factors associated with HRQoL. Statistical analyses were performed using STATA version 14 and statistical significance was determined at *p* < 0.05.

**Results:**

A total of 105 patients with hemophilia participated in the study, with a mean (standard deviation (SD) age of 21.09 (± 7.37] years. The median (IQR) EQ-5D-5L utility and EQ-VAS scores were 0.86 (0.59–0.91) and 75 (60.0–80.0), respectively. Age was significantly negatively associated with the EQ-5D-5L utility index and EQ-VAS (β = -0.020, 95 CI = -0.034, -0.007) and β = -0.974, 95% CI = -1.72, 0.225), respectively. The duration since hemophilia diagnosis (β-0.011, 95% CI, 0.001–0.023) and living out of Addis Ababa (β = -0.128, 95% CI, -0.248-, -0.007) were also significantly negatively associated with the EQ-5D-5L utility index..

**Conclusion:**

The median EQ-5D-5L utility and EQ-VAS scores of patients with hemophilia were 0.86 (0.59–0.91) and 75 (60.0–80.0), respectively. Older age, living far from the Hemophilia Treatment Center (HTC), and longer duration since diagnosis were significantly negatively associated with HRQoL. HRQoL may be improved by providing factor concentrates, decentralizing HTCs in different parts of the country, increasing awareness of bleeding disorders among health professionals, and providing psychosocial support to affected patients.

**Supplementary Information:**

The online version contains supplementary material available at 10.1186/s40780-023-00326-6.

## Introduction

Hemophilia is a rare genetic condition that is often overlooked in many countries, especially in low-income and middle-income countries [1]. It is a clotting disorder in which clotting factors are deficient VIII (hemophilia A) or IX (hemophilia B), which can cause internal bleeding in joints and muscles. This disorder mostly occurs in males. Although figures are inconsistent, an estimated one million men worldwide have hemophilia, and the incidence is estimated to be approximately one in 10,000 cases of hemophilia [2–4]. In Ethiopia, approximately 450 patients with hemophilia are treated at the Hemophilia Treatment Center (HTC). Frequent and high rates of joint bleeding can impair mobility and lead to permanent disability and premature death, posing significant economic and health burdens on patients, families, and the healthcare system. In addition, hemophilia symptoms and their complications negatively impact a patient's HRQoL [5–7].

Health-related quality of life is a patient-reported outcome measure that evaluates the effect of a disease, its complications, and disease management on a patient's health status. It can provide information about a person’s general health state, focusing on psychosocial, physical, and their respective impacts on the health status of the patient, which is affected by an individual’s beliefs, perceptions, experiences, and expectations [8, 9]. As such, HRQL is an essential measure of the impact of health interventions on patient-reported outcomes [10, 11]. In addition to drug therapy for hemophilia patients, evaluation of patient-reported problems provides information on the overall well-being of the patient and the impact of the disease on the patient [12, 13]. Although objective assessment metrics are used in pharmacological management to measure clinical outcomes, patient-reported outcome measures have not been addressed, which is no exception for hemophilia patients [14]. Most hemophilic patients experience repeated joint hemorrhage that causes unbearable joint pain, impairment, and deformity, resulting in a significant negative impact on their HRQoL [15]. This illustrates that clinical assessment alone may not be sufficient to characterize the morbidity associated with hemophilia, and it is necessary to measure patient-reported HRQoL and the factors associated with reduced HRQoL [16, 17]. Previous studies identified two target joint involvement, the frequency of joint pain, and the occurrence of joint surgery as independent predictors of lower HRQoL in hemophilia patients [17, 18]. Similarly, the presence of arthropathy, frequency of bleeding episodes, and mode of treatment administration (on-demand vs. prophylaxis) significantly decreased HRQoL in patients with hemophilia [16, 19–22]. In contrast, the use of prophylactic treatment was positively associated with HRQoL [7, 22–24].

A variety of generic and disease-specific tools have been developed to measure HRQoL for various diseases [25–27]. Among these tools, the European Quality of Life five-dimension, five-level scale questionnaire (EQ-5D-5L) is a widely used generic, preference-based, multi-attribute utility instrument to measure the impact of disease on HRQoL, and has been utilized for health technology assessment in many jurisdictions [28–30]. It measures the health state of patients by generating a single summary utility value that reflects how good or bad a health state is based on the preferences of the general population. The weight generated is a valuable indicator of the impact of disease and treatment and may help determine the allocation of resources for various health strategies [30]. However, in Ethiopia, HRQoL in patients with hemophilia has not been measured in any study. Hence, this study aimed to measure HRQoL and its associated factors in Ethiopian hemophilic patients using the EQ-5D-5L instrument at the HTC of Tikur Anbessa Specialized Hospital (TASH). The findings of this study can be utilized by clinicians and policymakers to develop tailored intervention strategies to enhance overall patient treatment outcomes.

## Methods

### Study design and setting

An institutional-based cross-sectional study was conducted between March and June 2023 among hemophilic patients at the HTC of TASH in Addis Ababa, Ethiopia. The hospital is the largest tertiary care teaching hospital in the country, with over 800 beds and over half a million patients annually. TASH has been the only HTC in the country for a long time. According to the hospital's Health Management Information System (HMIS) data, there are 452 hemophilia patients registered in the TASH HTC.

### Patient recruitment and data collection procedures

All patients with hemophilia visiting the HTC comprised the source population, while the study population included all active patients aged > 15 years and those who were willing to participate and fulfilled the eligibility criteria during the data collection period. Patients who had been diagnosed with hemophilia within the last three months and were critically ill, and unable to participate were excluded from the study. Consequently, we included 105 eligible patients with hemophilia who visited the HTC during the data collection period, forming the sample size. Participants were recruited using a consecutive sampling method. Before actual data collection, the research team prepared and pretested the data collection tools. Data collectors were trained on how to use the data collection instruments, how to approach the study participants, collect clinical data from the hospital's iCare electronic system, and maintain the quality of the information collected. The data collection tool had three parts: patient sociodemographic characteristics obtained directly from the patient (age, sex, marital status, education level, employment status, place of residence, and health service charge), patient clinical characteristics obtained from the patient medical record (type of hemophilia, target joint, treatment strategies, type of product used), and the EQ-5D-5L to measure the HRQoL of the patients.

### EQ-5D-5L tool

The instrument has five dimensions and five levels, four of which are physical and one is psychological [27, 30]. A validated version of the EQ-5D-5 L was used for the interviews [31]. The tool consists of two components: the EQ-5D-5L descriptive short questionnaire and the European Quality Visual Analog Scale (EQ-VAS). In the first part of the instrument, patients select the statement most reflective of their health state in the descriptive system, which has five dimensions (mobility, self-care, usual activities, pain or discomfort, and anxiety or depression). The severity of the problems is represented by five levels: no problems, slight problems, moderate problems, severe problems, and extreme problems. We asked the participants to choose one level that reflected their health state on the interview date for each of the five dimensions. The EQ-VAS is used for the subjective assessment of one’s current health state from the patient’s perspective. Using this scale, each patient self-rated his or her health status on a vertical scale ranging from zero (worst health one can imagine) to 100 (best health one can imagine).

### Statistical analysis

The sociodemographic and clinical characteristics of the patients were presented using descriptive statistics (median, mean, percentage, and frequency). The χ2 test was used to illustrate the differences in the percentage of reported problems. The severity of each aspect of the EQ-5D-5L descriptor system was reported using five levels to determine the proportion of reported health problems in different subgroups (1 = no problems to 5 = serious problems). The EQ-5D-5L utility score was computed using disutility coefficients (decrements in utility) obtained from the Ethiopian general population using a hybrid regression model (29). As the EQ-5D-5L utility and EQ-VAS scores were non-normally distributed (Shapiro–Wilk test, *p* < 0.05), we presented the median [interquartile range (IQR)] scores. We applied the Kruskal–Wallis and Mann–Whitney U tests to determine the difference in EQ-5D-5L and EQ-VAS utility scores between patient groups. A multivariate Tobit regression model was used to identify associated factors of HRQoL. Some covariates were not entered into the regression models because of the small number of responses. We censored the utility score at one and the EQ-VAS score at 100. Statistical analyses were performed using STATA Version 14. All statistical tests were performed using a level of significance of *p*-value < 0.05.

### Ethical consideration

Ethical clearance was obtained from the Ethical Review Committee of the School of Pharmacy, College of Health Sciences, Addis Ababa University, Ethiopia (ERB/SOP/477/15/2023). Before data collection, a written permission letter was obtained from the HTC of TASH. The participants were provided with a clear explanation of the objectives of the study. After obtaining written informed consent from each participant, information was collected and taken from their family or legal guardian for participants who were between 15–18 years old. The study participants were provided with the right to refuse or interrupt their participation at any time and the opportunity to ask questions about the study. For obscurity, the participant’s name was not used at the time of data collection, all other personnel information was kept entirely obscure, and confidentiality was assured throughout the study period.

## Results

### Sociodemographic characteristics of the patients

A total of 105 patients with hemophilia who have attended the HTC of TASH were included in the present study. All patients were male with a mean (standard deviation (SD) age, of 21.09 (+ 7.37) years). Of the participants, 95 (90.5%) were unmarried and 58.1% of the patients live in Addis Ababa. Nearly all patients (92.4%) of them live with their families, and 92 (87.6%) of them get health services at the centre (Table 1).

**Table 1:** Socio-demographic characteristics of hemophilia patients

Variables	N (%)
Age	
15–24 years	74 (70.5)
25–34 years	26 (24.8)
≥ 35 years	5 (4.8)
Marital status	
Single	95 (90.5)
Married	10 (9.5)
Residence	
Addis Ababa	61(58.1)
Out of Addis Ababa	44 (41.9)
Educational status	
Unable to write and read	3 (2.9)
Primary school (1–8)	36 (34.3)
Secondary school (9–12)	36 (34.3)
Diploma	7 (6.7)
Degree and above	23 (21.9)
Religion	
Orthodox	58 (55.2)
Muslim	24 (22.9)
Protestant	21 (20.0)
Catholic	2 (1.9)
Employment status	
Student	68 (64.8)
Employed	9 (8.6)
Self-employed	5 (4.8)
Farmer	2 (1.9)
Not working/unemployed	21 (20.0)
With whom do you live?	
Family	97 (92.4)
Other (in a dormitory, police camp, etc.)	5 (4.8)
Alone	3 (2.9)
Payment method	
Free	92 (87.6)
Health insurance	9 (8.6%)
Out of pocket	4 (3.8)

### Clinical characteristics of hemophilia patients

Among the hemophilic patients, 57 (54.3%) of them had visited the emergency room, and about a quarter of them had been admitted to the hospital in the previous year. The majority 81(77.1%), had heavy bleeding in the past year which occurred four times in 74 patients. Nearly a third (71.4%) of them had a family history of hemophilia,76.2% of patients received factor VIII, and 76 (72.4%) patients had hemophilia A. Joint involvement was reported in 88 patients where the knee involvement (70.4%) was the most affected joint. In about half of the patients (51.4%), hemophilia was diagnosed between 11 and 19 years of their age (Table 2).

**Table 2:** Clinical characteristics of patients with hemophilia

Clinical Variables	N (%)
Visiting the emergency department in last year	
No	48 (45.7)
Yes	57 (54.3)
Hospitalization in the last year	
No	86 (81.9)
Yes	19 (18.1)
Heavy bleeding in the last year	
No	24 (22.9
Yes	81 (77.1)
Number of bleedings in last year	
None	22 (21.2)
One- three times	8 (7.7)
≥ four times	74 (71.2)
Family history of Hemophilia	
No	30 (28.6)
Yes	75 (71.4)
Current hemophilia treatment	
Factor VIII	80 (76.2)
Factor IX	23 (21.9)
Plasma and Tranexamic acid	2 (1.9)
Hemophilia type	
Hemophilia A	76 (72.4)
Hemophilia B	29 (27.6)
Target joint involvement	
No	20 (19.0)
Yes	85 (81.0)
Number of targets joint involvement	
One	49 (46.7)
Two and above	39 (37.2)
Location of target joint	
Knee	74 (70.4)
Hip	16 (15.2)
Elbow	26 (24.7)
Shoulder	12 (11.4)
Ankle	10 (9.5)
Wrist	8 (7.6)
Time since diagnosis	
≤ 10 years	24 (22.9)
11–19 years	54 (51.4)
≥ 20 years	27 (25.7)

### Distribution of EQ-5D-5L descriptive dimensions

**Fig. 1:**
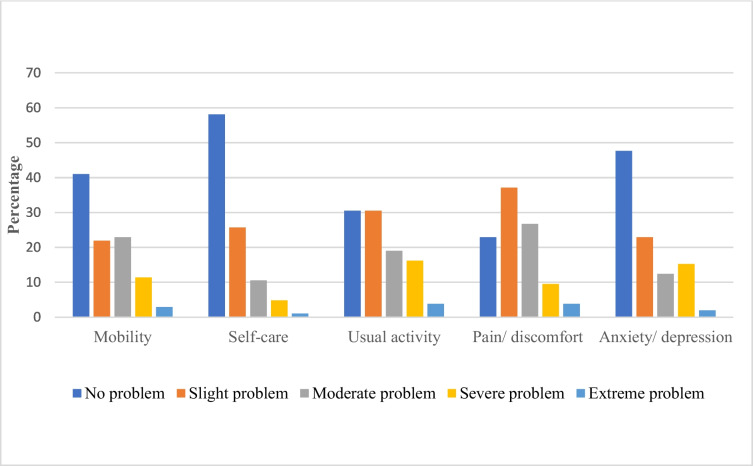
Self-reported health problems using the EQ-5D-5L descriptive dimensions in patients with hemophilia

In the mobility domain of the EQ-5D-5L, 41% of the patients reported having no problems. In the remaining different levels of problems were reported, with moderate problems (22.9%) being the most documented. While 58.1% had no problem concerning self-care, (25.7%) had slight problems, and only 1% had extreme problems with clothing and washing. On the other hand, 30.5% of the respondents had no problems associated with usual activities, such as schooling and work. In (22.9%) of cases, no pain or discomfort was reported, (37.1%) of respondents complained of slight problems, the remaining (26.7%) had moderate problems, (9.5%) had severe problems, and (3.8%) complained of extreme problems. Regarding anxiety or depression, (47.6%) were not depressed or anxious, (22.9%) had slight problems, and moderate problems were reported by (12.4%) had moderate problems. The remaining (15.2%) and (1.5%) patients had severe and extreme problems, respectively (Fig. 1).

### Percentage of self-reported problems in EQ-5D-5L dimension

Among the five EQ-5D-5L dimensions, pain/discomfort was the most frequent (77.1%) reported problem followed by usual activity (69.5%) (Supplementary file). On the contrary, self-care (41.9%) was the lowest reported problem. Patients in the 15–24 age group had more problems in usual activity (44.8%) and pain/discomfort (49.5%). Additionally, patients who had experienced major bleeding in the past year had more frequent problems with mobility (49.5%), self-care (34.3%), usual activities (57.1%), pain/discomfort (61.0%), and anxiety/depression (41.9%), respectively. The detailed self-reported health problems in each domain are depicted in Supplementary file 1.

### EQ-5D-5L index value and EQ-VAS score

The median (IQR) EQ-5D-5L utility and EQ-VAS scores were 0.86 (0.59–0.91) and 75 (60.0–80.0), respectively with mean of 0.71 ± 0.31(SD) and 69.1 ± 17.1(SD). The median EQ-5D-5L index was significantly higher in patients aged 15–24 years than in those aged greater than 35 years (0.60 vs. 0.31) (*p* = 0.001). There were significant differences in EQ-VAS scores among patients who get medical services for free compared to those who use community-based health insurance (*p* = 0.005). Otherwise, there were no significant differences among different socio-demographic and clinical characteristics on both median EQ-5D-5L utility and EQ-VAS score in this study (Table 3). Patients in the study who lived alone had lower mean utility index (32.83) and EQ-VAS scores (36.17) than those who lived with family members or others.

**Table 3:** Median (IQR) differences of EQ-5D-5L utility and EQ-VAS scores with the different patient characteristics

Variables	EQ-5D-5L index median score (IQR)	Mean rank	*p*-value	EQ-VAS Median score (IQR)	Mean rank	*p*-value
**Age category**						
15–24	0.89 [0.77–0.93]	60.0	0.001*	75[60.0–85.0]	58.24	0.023*
25–34	0.63 [0.48–0.84]	37.22		62[55.0–75.0]	40.71	
> 35	0.73 [0.32–0.80]	31.4		75[35.0–77.5]	39.40	
**Marital status**						
Unmarried	0.86 [0.64–0.91]	54.66	0.087	75[60.0–80.0]	53.85	0.374
Married	0.65 [0.52–0.85]	37.35		70[48.7–76.2]	44.90	
**Education status**						
Unable to write and read	0.93 [0.86–1.00]	81.0	0.597	60[55.0–70.0]	32.67	0.679
Primary school (1–8)	0.86 [0.56–0.92]	53.3		75[60.0–80.0]	52.50	
Secondary school (9–12)	0.85 [0.44–0.91]	51.31		77[60.0–85.0]	57.14	
Diploma	0.80 [0.54–0.93]	49.14		65[50.0–80.0]	47.57	
Degree and above	0.84 [0.61–0.91]	52.7		75[60.0–80.0]	51.61	
**Residence**						
Addis Ababa	0.87 [0.73–0.91]	57.36	0.084	75[60.0–82.5]	57.34	0.083
Out of Addis Ababa	0.78 [0.41–0.91]	46.95		70[50.0–78.7]	46.98	
**Religion**						
Orthodox	0.85 [0.54–0.92]	52.66	0.499	75[60.0–85.0]	57.43	0.166
Muslim	0.85 [0.72–0.89]	52.5		70[60.0–78.7]	46.06	
Protestant	0.87[0.0.69–0.93]	57.93		70[57.5–80.0]	49.21	
**Employment status**						
Employed	0.77 [0.57–0.86]	44.06	0.148	75[55.0–77.5]	48.44	0.060
Self-employed	0.78 [0.31–0.90]	43.6		55[42.5–85.0]	41.30	
Student	0.88 [0.71–0.92]	58.54		75[60.0–85.0]	59.18	
Not working	0.70 [0.38–0.91]	43.3		60[50.0–72.5]	37.83	
**Living with**						
With Family	0.85 [0.81–0.91]	53.29	0.461	75[60.0–80.0]	53.94	0.499
With other people	0.87 [0.64–0.91]	59.4		75[50.0–77.5]	44.80	
Alone	0.33 [0.33–0.91]	32.83		65[45.0–75.0]	36.17	
**Health service charge**						
Free	0.85 [0.61–0.91]	52.9	0.692	75[60.0–80.0]	55.29	0.005*
Out of pocket	0.91 [0.53–0.93]	64.5		77[75.0–83.7]	68.63	
Community-based health insurance	0.86 [0.43–0.91]	48.89		55[42.5–60.0]	22.67	
**Visiting emergency in the past year**						
No	0.87 [0.62–0.93]	57.06	0.209	75[56.7–85.0]	54.43	0.657
Yes	0.81 [0.54–0.89]	49.58		75[60.0–80.0]	51.80	
**Hospitalization in the past year**						
No	0.85 [0.57–0.91]	53.43	0.758	75[60.0–80.0]	54.84	0.184
Yes	0.86 [0.54–0.91]	51.05		60[45.0–80.0]	44.66	
**Time since diagnosis**						
< 10 years	0.89 [0.72–0.93]	61.67	0.195	75[56.2–85.0]	56.15	0.531
11–19 years	0.86 [0.60–0.91]	52.49		75[60.0–80.0]	54.36	
> 20 years	0.78 [0.50–0.87]	46.31		65[60.0–80.0]	47.48	
**Heavy bleeding in the past year**						
No	0.90 [0.81–0.93]	62.4	0.085	77[55.0–85.0]	57.96	0.361
Yes	0.83 [0.54–0.91]	50.22		75[60.0–80.0]	51.53	
**Number of bleedings**						
None	0.90 [0.81–0.93]	61.52	0.124	77[55.0–85.0]	56.65	0.249
1-3times	0.85 [0.57–0.91]	52.12		75[60.0–80.0]	53.66	
> 4times	0.65 [0.27–0.86]	36.63		60[36.2–78.7]	36.38	
**Family history**						
**No**	0.90 [0.81–0.93]	60.8	0.097	80[60.0–81.2]	59.95	0.137
**Yes**	0.85 [0.57–0.91]	49.88		70[55.0–80.0]	50.22	
**Current treatment**						
Factor 8	0.86 [0.61–0.92]	54.72	0.532	75[60.0–90.0]	54.09	0.609
Factor 9	0.78 [0.58–0.89]	46.72		75[55.0–80.0]	50.83	
**Hemophilia type**						
Hemophilia A	0.85 [0.61–0.91]	53.09	0.963	70[55.0–80.0]	50.05	0.105
Hemophilia B	0.86 [0.54–0.89]	52.78		75[70.0–80.0]	60.74	
**Target joint involvement**						
**No**	0.86 [0.40–0.89]	48.78	0.490	72[60.0–83.7]	52.63	0.951
**Yes**	0.85 [0.61–0.92]	53.99		75[60.0–80.0]	53.09	
**Time since treatment started**						
** < 10 years**	0.89 [0.65–0.93]	59.44	0.200	75[52.5–85.0]	55.31	0.398
**11–19 years**	0.87 [0.64–0.91]	54.32		75[60.0–80.0]	55.38	
> 20 years	0.78 [0.50–0.87]	44.63		65[55.0–80.0]	46.19	

*EQ-5D-5L* EuroQoL life questionnaires five dimensions with five levels, *IQR* interquartile range, *EQ-VAS* Quality of Life Group’s Visual Analog Scale

^*^indicates *p*-value with a significant statistical association

### Factors associated with HRQoL

The multivariable Tobit regression model (Table 4) showed that age was significantly negatively associated with the EQ-5D-5L utility index and EQ-VAS (β = -0.020, 95 CI = -0.034, -0.007) and β = -0. 974, 95% CI = -1.72, 0.225), respectively. Duration since hemophilia diagnosis (β-0.011, 95 CI%, 0.001–0.023) and living out of Addis Ababa (β = -0.128 95 CI%, -0.248-, -0.007) were also significantly negatively associated with the EQ-5D-5L utility index. Conversely, the level of education, treatment taken, number of joints involved, marital status, and emergency department visit status in the past year were not significantly associated with both the EQ-5D-5L index score and EQ-VAS in this study.

**Table 4:** Predictors of HRQoL in patients with hemophilia

Variables	EQ-5D-5L index score				EQ-VAS			
	**β-Coeff. [95%CI]**	**SE**	**t**	***P*-value**	**β-Coeff. [95%CI]**	**SE**	**t**	***P*-value**
**Since the diagnosis of hemophilia**	-0.011[0.001–0.023]	-0.006	2.05	0.043*	-0.265[-1.22, 0.686]	0.479	-0.55	0.582
**Age**	-0.020 [-0.034-, -0.007]	0.007	-3.05	0.003*	-0.974 [-1.72, 0.225]	0.377	-2.58	0.011*
**Place residence (ref = Addis Ababa)**								
Out of Addis Ababa	-0.128 [-0.248-, -0.007]	0.061	-2.10	0.038*	-5.12 [-11.87, 1.62]	3.398	-1.51	0.135
**Level of education (ref = illiterate**								
Primary school	-0.185 [-0.554, 0.185]	0.186	-0.99	0.324	1.45 [-2.50, 5.39]	1.99	0.73	0.467
Secondary school	-0.192 [-0.568, 0.184]	0.189	-1.01	0.314	0.471 [-3.77, 4.71]	2.13	0.22	0.345
Higher education	-0.135 [-0.529, 0.259]	0.199	-0.68	0.498	-0.017[-0.092,0.056]	0.037	-0.48	0.634
**Employment status (ref = unemployed)**								
Employed	0.124 [-0.113, 0.3621]	0.119	1.04	0.302	3.99 [-9.67, 17.7]	6.88	0.58	0.563
Student	-0.008 [-0.185, 0.170]	0.089	-0.09	0.931	5.32 [ -4.75, 15.4]	5.07	1.05	0.297
**Treatment taking (ref = factor 8)**								
Factor 9	-0.059 [-0.200, 0.082]	0.071	-0.83	0.407	3.01 [ -5.11, 11.1]	4.08	0.74	0.464
Plasma	-0.170 [-0.581, 0.240]	0.207	-0.82	0.412	-11.56 [ -35.3,12.2]	11.9	-0.97	0.337
**Total joint involvement (ref = < 2)**								
Greater than 2	-0.003 [-0.060, 0.053]	0.028	-0.11	0.913	0.15 [ -3.09, 3.39]	1.63	0.09	0.926
**Emergency in last year (ref = no)**								
Yes	-0.015 [-0.128–0.097]	0.057	-0.27	0.786	-1.63 [-8.66- 5.38]	3.53	-0.46	0.644
**Marital status**								
Unmarried	0.05 [ -0.23- 0.334]	0.14	0.36	0.719	5.37 [-8.08- 18.8]	6.77	0.79	0.430

^*^p-value < 0.05. *CI* Confidence interval, *EQ-5D-5L* EuroQoL life questionnaires five dimensions with five levels, *EQ-VAS* Quality of Life Group’s Visual Analog Scale Ref, Reference. * indicates *p*-value with a significant statistical association

## Discussion

The HRQoL measurements are frequently used in clinical practice and research to assess disease burden and treatment outcomes. Thus, the purpose of this study was to evaluate HRQoL and associated factors in hemophilia patients in an Ethiopian HTC. In all five dimensions of the EQ -5D-5L, pain/discomfort was the most frequently (77.1%) mentioned problem, and pain/discomfort and usual activities were the most common dimensions in which 'extreme' problems were reported in our study. A study in five European countries reported target joints, pain/discomfort, and anxiety/depression in hemophilia patients [6]. The second most common problem reported was problems performing usual activities, where hemophilia is a reason for most to drop out of education or miss school days and quit work because of the pain, accidental bleeding emergencies, and subsequent hospitalizations. Conversely, compared to the rest of the EQ-5D-5L dimensions, self-care, and anxiety/depression were reported as 'no problem’ in 58.1% and 47.6% of patients, respectively in the present study.

The mean utility in our study was 0.71 (± 0.31), which was comparable to a global multicenter study with a mean utility value of 0.72 (± 0.23) [32]. However, the mean utility value in this study was lower than Germany's 0.90 (± 0.12), France's 0.75(± 0.28), and Italy's 0.85 (± 0.12) [6] findings, and the mean utility value of 0.92 for the Ethiopian general population [31]. This could be due to a lack of adequate healthcare infrastructure and a shortage of trained professionals to provide care for patients with hemophilia, and also competing government healthcare priorities, lower visibility of hemophilia patients in the healthcare system, and a lack of factor concentrates, all of which have a direct impact on HRQoL. Nevertheless, the mean utility value obtained in in the present study was higher than that of the China study which reported a mean utility value of 0.51 (± 0.34) in hemophilia patients [33]. Likewise, the mean EQ-VAS score was 69.1(± 17.1) in our study, which is in line with the prospective multicentre study 73.5 (± 20.1) [32] report). These discrepancies in the mean EQ-VAS score values could be explained by changes in patients’ profiles, sociocultural perspectives, and disparities in access to medical care. Patients taking Factor VIII (54.09%) had a relatively better EQ-VAS score than those taking Factor IX (50.83%) and plasma/tranexamic acid (34.25%). This may be because FVIII concentrates are relatively frequently available in the HTC and its administration may have minimized bleeding, joint pain, swelling, painful movements, or difficulty walking as compared to patients with FIX deficiency where treatment is only rarely available.

The present study identified older age, residence outside Addis Ababa, and time since diagnosis associated with negatively with the HRQoL. In patients living in Addis Ababa, fewer problems were reported than those who lived outside of Addis Ababa in all EQ-5D-5L dimensions, indicating that their residential status was crucial. This could be due to patients living outside of Addis Ababa having less access to healthcare services, transportation issues for quick visits to the emergency department and HTC when bleeding occurs, and ultimately affecting their HRQoL. Similarly, patients admitted to the hospital and those who visited the emergency department frequently had more difficulties with mobility, self-care, normal activities, pain/discomfort, and anxiety/depression.

Similar to a study conducted in Heilongjiang Province of China that reported a significant difference in the mean EQ-5D utility score by age group (≥ 25 vs < 25 years, -0.157 (-0.282, -0.032, *p* = 0.014)) [17], we found statistically significant differences in EQ-5D-5L index and EQ-VAS scores among participants with different age categories. In addition, EQ-VAS scores showed statistically significant differences between different medical service payment methods, and a similar association was shown in Chinese studies [17, 34]. The present study identified that age, living longer with hemophilia, and coming from out of Addis Ababa as significant negative predictors of HRQoL. The decline of HRQoL in older individuals could be attributed to increased deterioration of physical functioning, which might increase the progression of joint damages and ultimately reduction in overall HRQoL. Likewise, lower HRQoL among hemophilic patients living with the disease for a longer time could be explained mainly due to the progression and worsening of the disease conditions and disability. Furthermore, hemophilic patients who came from outside of Addis Ababa had lower HRQoL, which might be attributed to that those patients travel a long distance to get the services to the capital city as it is the only hemophilic center in the country and this could be the reason why those patients had lower HRQoL. The government should expand HTCs in different corners of the country and stakeholders shall also be involved in public awareness about hemophilia. This would be the best remedy to enhance patients' HRQoL which in turn increases patient satisfaction.

### Limitations of the study

Recruiting participants with rare diseases was challenging and limited the sample size in our study as there were only 452 patients registered since the centre's establishment. The study included only patients who attended the HTC during the study period and those who did not seek care at the hospital or who were unable to attend the clinic may have different experiences in their HRQoL. Being a cross-sectional design in its nature, establishing causal relationships or the assessment of changes in HRQoL over time was not allowed. The study was conducted over a relatively short period which may not be possible to capture seasonal variations or long-term changes in HRQoL among hemophilia patients. Being uncircumcised may also have a negative impact on the HRQoL of our patients especially on the anxiety/ depression domain of EQ-5D-SL which we didn’t study.

## Conclusions

In general, we found that living with hemophilia, had a profound negative effect on patients’ HRQoL. Most patients reported problems with pain/discomfort and performing usual activities. The median EQ-5D-5L utility and EQ-VAS scores in hemophilia patients were 0.69 and 71, respectively. Older age, living far from the HTC, and longer periods since diagnosis were significantly negatively associated with HRQoL. Therefore, future intervention efforts aimed at improving HRQoL in hemophilia patients should be designed to address these factors. The HRQoL of hemophilia patients in low-income countries may be improved by expanding treatment centers in different parts of the county enhancing healthcare systems and providing psychosocial support.

### Supplementary Information

**Additional file 1.** Percentage of self-reported health problems among patients with hemophilia.

## Data Availability

The datasets used and/or analyzed during the current study are available from the corresponding author upon reasonable request**.**

## Abbreviations

EQ-5D-5L: Euro Quality of Life Group’s 5-Domain Questionnaires 5 Levels
EQ-VAS: Quality of Life Group’s Visual Analog Scale
HTC: Hemophilia Treatment Centre
HRQoL: Health-Related Quality of Life
TASH: Tikur Anbessa Specialized Hospital
WFH: World Federation of Hemophilia
WHO: World Health Organization
