# Supplementary material for: Health-related quality of life and its associated factors among hemophilia patients: experience from Ethiopian Hemophilia Treatment Centre
Source: J Pharm Health Care Sci. 2024 Jan 2;10:3. doi: 10.1186/s40780-023-00326-6 (PMC10762823; doi:10.1186/s40780-023-00326-6)
Supplement: Supplementary file 1 — Additional file 1. Percentage of self-reported health problems among patients with hemophilia. [file 40780_2023_326_MOESM1_ESM.docx]

**Percentage of self-reported health problems among patients with hemophilia**

| Variables | **Percentage of reported problems** | | | | |
| --- | --- | --- | --- | --- | --- |
|  | Mobility | Self-care | Usual activity | Pain/  discomfort | Anxiety/ Depression |
| **Participants** | 59 | 41.9 | 69.5 | 77.1 | 52.4 |
| Age category | | | | | |
| 15-24 | 32.4 | 26.7 | 44.8 | 49.5 | 31.4 |
| 25-34 | 21.9 | 14.3 | 21 | 22.9 | 19.0 |
| >35 | 4.8 | 1 | 3.8 | 4.8 | 1.9 |
| Marital status | | | | | |
| Single | 49.5 | 36.2 | 60.0 | 68.6 | 46.7 |
| Married | 9.5 | 5.7 | 9.5 | 8.6 | 5.7 |
| Educational status | | | | | |
| Unable to read and write | 0 | 0 | 0 | 1.9 | 1.0 |
| Primary school (Grades 1-8) | 16.2 | 13.3 | 25.7 | 26.7 | 16.2 |
| Secondary school (Grade 9-12) | 22.9 | 16.2 | 22.9 | 24.8 | 14.3 |
| Diploma/TVET | 3.8 | 2.9 | 5.7 | 5.7 | 5.7 |
| Degree and above | 16.2 | 5.7 | 15.2 | 18.1 | 15.2 |
| Residence | | | | | |
| Addis Ababa | 33.3 | 23.8 | 37.1 | 42.9 | 28.6 |
| Out of Addis Ababa | 25.7 | 18.1 | 32.4 | 34.3 | 23.8 |
| Religion | | | | | |
| Orthodox | 31.4 | 19.0 | 37.1 | 39.0 | 28.6 |
| Muslim | 15.2 | 12.4 | 17.1 | 21.0 | 12.4 |
| Protestant | 10.5 | 9.5 | 14.3 | 15.2 | 9.5 |
| Catholic |  |  |  |  |  |
| Employment status | | | | | |
| Employed | 7.6 | 5.7 | 7.6 | 7.6 | 6.7 |
| Self-employed | 4.8 | 1.0 | 4.8 | 3.8 | 2.9 |
| Student | 32.4 | 24.8 | 42.9 | 46.7 | 30.5 |
| Farmer | 1.9 | 0.0 | 1.9 | 1.9 | 1.0 |
| Not working/ unemployed | 12.4 | 10.5 | 12.4 | 17.1 | 11.7 |
| With whom do you live? | | | | | |
| Family | 54.3 | 39.0 | 65.7 | 69.5 | 47.6 |
| Other people (in a dormitory, police camp, etc) | 2.9 | 1.9 | 2.9 | 4.8 | 1.9 |
| Alone | 1.9 | 1.0 | 1.0 | 2.9 | 2.9 |
| Payment method | | | | | |
| Free | 53.3 | 38.1 | 61.9 | 66.7 | 45.7 |
| Out of pocket | 1.0 | 1.9 | 2.9 | 2.9 | 1.0 |
| Health insurance | 4.8 | 1.9 | 4.8 | 7.6 | 5.7 |
| Visiting of emergency in the past year | | | | | |
| No | 23.8 | 15.2 | 29.5 | 33.3 | 20.0 |
| Yes | 35.2 | 26.7 | 40.0 | 43.8 | 32.4 |
| Hospitalized for hemophilia in the past year | | | | | |
| No | 47.6 | 35.2 | 58.1 | 61.9 | 44.8 |
| Yes | 11.4 | 6.7 | 11.4 | 15.2 | 7.6 |
| Time since hemophilia diagnosis | | | | | |
| ≤10 years | 10.5 | 5.7 | 13.3 | 15.2 | 10.5 |
| 11-19 years | 29.5 | 25.7 | 39.0 | 40.0 | 23.8 |
| ≥20 years | 19.0 | 10.5 | 17.1 | 21.9 | 18.1 |
| Heavy bleeding in the past year | | | | | |
| No | 9.5 | 7.6 | 12.4 | 16.2 | 10.5 |
| Yes | 49.5 | 34.3 | 57.1 | 61.0 | 41.9 |
| Bleeding in the past year | | | | | |
| None | 8.7 | 7.7 | 11.5 | 15.4 | 9.6 |
| 1-3 times | 44.2 | 30.8 | 51.9 | 55.8 | 39.4 |
